# Editorial Comment: The effects of pregabalin, solifenacin and their combination therapy on ureteral double-J stent-related symptoms: A randomized controlled clinical trial

**DOI:** 10.1590/S1677-5538.IBJU.2022.02.04

**Published:** 2022-03-01

**Authors:** Alexandre Danilovic

**Affiliations:** 1 Faculdade de Medicina da Universidade de São Paulo Hospital das Clínicas Departamento de Urologia São Paulo SP Brasil Departamento de Urologia, Hospital das Clínicas, Faculdade de Medicina da Universidade de São Paulo, São Paulo, SP, Brasil

Siavash Falahatkar 1, Mohammadreza Beigzadeh 1, Gholamreza Mokhtari 1, Samaneh Esmaeili 1, Ehsan Kazemnezhad 1, Atiyeh Amin 1, et al.

Int Braz J Urol. May-Jun 2021;47(3):596-609.

## COMMENT

Ureteral stents are frequently used in urology practice, particularly following ureteroscopy for urinary stone treatment (1). However, urinary stent-related symptoms are among the most bothersome symptoms affecting patient's quality of life (2, 3). Stent-related symptoms are usually worse during the first four days after surgery, with a peak of medication use on day 1 after placement (4). Sometimes, these symptoms are so limiting that the patient usually refers the treatment for urinary stone as stent placement. Stent composition, design and size, and medical therapies have been studied to mitigate patient's suffering.

Some strategies have been proved to reduce stent-related symptoms. Silicone composed stents are associated with less patient discomfort (5, 6). A novel intraureteral stent placement was associated with less discomfort than conventional stent placement (7). Other studies found that stent-related patient symptoms increase with the diameter of the stents. Therefore, smaller diameter stents should be preferred (8, 9). Stent-related symptoms may be significantly mitigated with drug monotherapy or combination of beta-3 adrenergic receptor agonist, alpha-blockers and anticholinergic (10–16).

Falahatkar et al. studied the effects of pregabalin, solifenacin and their combination therapy on urinary stent-related symptoms in a randomized controlled clinical trial (17). Patients were randomly allocated into four groups: pregabalin 75 mg BID (N=64), solifenacin 5 mg once a day (N=64), pregabalin 75 mg BID and solifenacin 5 mg once a day (N=64), and no medication (N=64). Ureteral Symptom Score Questionnaire (USSQ) was used to compare groups at 2 and 4 weeks after discharge from hospital (18). Authors reported significant beneficial effects in all indexes of USSQ only for combined pregabalin and solifenacin therapy over control group. Reported side effects were mild for all studied groups. Lack of a placebo arm and application of USSQ only at 2 and 4 weeks after discharge from hospital are some of the limitations of this study.

Urinary stent-related symptoms should not be overlooked and could be relieved by an adequate stent selection and a combination of postoperative medical therapy.
